# Compensatory gain based on lysine level in finishing pigs after being fed lysine deficient 97% corn diets for 3 or 6 wk

**DOI:** 10.1093/tas/txad095

**Published:** 2023-08-08

**Authors:** Chloe S Hagen, Joel D Spencer, Greg T Krahn, Laura L Greiner

**Affiliations:** Department of Animal Science, Iowa State University, Ames, IA 50010, USA; United Animal Health, Sheridan, IN 46069, USA; Department of Animal Science, Iowa State University, Ames, IA 50010, USA; Department of Animal Science, Iowa State University, Ames, IA 50010, USA

**Keywords:** compensatory growth, lysine, pig market

## Abstract

The objective of this experiment was to evaluate increasing the concentration of lysine on the compensatory gain of finishing pigs during their recovery period after being fed a 97% corn holding diet for 3 or 6 wk. One thousand six hundred and eighty pigs with a starting body weight of 73.5 ± 2.2 kg were blocked by starting body weight and assigned to a nested arrangement. Twenty replicates of seven treatments were comprised of two restriction lengths [3 weeks (3 wk) vs. 6 weeks (6 wk)], and three lysine concentrations during recovery (Lys:ME same as control:100; control + 10%: 110; control + 20%: 120) plus one control (CONT) that remained nutrient unrestricted. Pen weight and feed intake were recorded on days 0, 21, 41, and at marketing. Whole pens were marketed when the pen average met 130 kg and carcass measurements were collected. Data were analyzed by pen with the fixed effects of restriction length and lysine within restriction length. Nutrient restriction lowered (*P* < 0.01) average daily gain (ADG) compared to control, with 1.2, 0.4, and 0.5 kg for control, 3 wk, and 6 wk treatments, respectively. Restricted pigs showed decreased feed intake while restricted. After the respective restriction period, pigs were allowed a recovery diet until market. Previously restricted pigs had 16.7% and 27.3% greater (*P* < 0.01) ADG over control pigs for 3 and 6 wk treatments, respectively, in the first 3-wk of recovery. The lysine concentration in the recovery diet impacted (*P* < 0.01) the ADG with pigs allowed the highest lysine concentration having a 10% greater ADG than pigs fed the lower Lys:ME concentrations, for both restriction treatments. The increase in ADG was not paralleled by an increase in feed intake over control, thus, there was an improvement (*P* < 0.01) in gain to feed ratio in the recovery period. Control pigs reached market weight (131.5 kg) on experiment day 49 while pigs fed corn diets for 3 wk or 6 wk were slower to market (57 and 69 days, respectively; *P* < 0.01). Restricted pigs had greater backfat (CONT: 1.47, 3 wk: 1.55, 6 wk: 1.65 cm; *P *< 0.01), and decreased loin depth (CONT: 7.32, 3 wk: 7.03, 6 wk: 6.61 cm, *P *< 0.02) which was also impacted (*P* < 0.01) by lysine concentration. In conclusion, the use of restrictive diets reduced ADG and increased days to market. The use of recovery diets in which the Lys:ME ratio was greater than control pigs, resulted in increased compensatory growth.

## Introduction

Compensatory gain is a phenomenon where pigs previously under nutritional restriction, grow at an accelerated rate during the re-feeding period (restriction removed) over pigs that had no previous nutrient restriction. Nutritional restriction can be accomplished by limiting feed intake or limiting the nutrient intake, thus slowing pig growth (Skiba, 2005). Slowing growth of finishing pigs was of interest in 2020 as COVID-19 caused disruptions in the marketability of market hogs and limited shackle space within harvest plants. Recommendations to reduce dietary crude protein and essential amino acids by feeding corn-only diets in the finishing phase were suggested to slow the growth of pigs and hold them at a marketable weight for a longer time (Hostetler, 2020; Patience and Greiner, 2020; Tokach et al., 2021). These recommendations were shown to be successful in commercial settings (Gabler et al., 2020; Helm et al., 2021; Rao et al., 2021). Studies evaluating growth after switching from these nutrient-restricted corn diets to an un-restricted diet showed compensatory gain (Gabler et al., 2020; Rao et al., 2021).

It has previously been suggested that the lysine requirement for pigs in a recovery or compensatory gain period after lysine restriction may be higher (Whang et al., 2003) than for pigs of similar age that did not face restriction. While the 97% corn-holding diet was surely restrictive in lysine, other amino acids such as methionine, threonine, and tryptophan were largely below requirements. Therefore, this study was not only a lysine restriction but also an amino acid restriction. However, due to the focus on increasing lysine in the recovery period, lysine is the amino acid discussed the most hereafter. The aim of this study was to evaluate the concept of increasing dietary lysine concentrations in the recovery period after a period of protein and amino acid restriction, as well as evaluate the effects of two restriction lengths on the ability of pigs to compensate. The restriction was accomplished by feeding a 97% corn diet, similar to diets used to slow market weight pigs in the face of the harvest-plant closures due to COVID-19. Based on the recommendation that nutrient requirements after a period of restriction may be increased, the objective was to determine how feeding diets with increased nutritional density impacts the growth performance of pigs after they have consumed a protein/lysine-restricted diet. The hypothesis was that compensatory gain will be increased for pigs fed diets with increased nutritional density after the restricted diets.

## Materials and Methods

All procedures in this experiment adhered to the guidelines for the ethical and humane use of animals for research and were approved by the Institutional Animal Care and Use Committee at Iowa State University (IACUC# 20-105). Pigs were housed in pens (0.62 m^2^ per pig) within two attached barns. Pens in one barn were equipped with one double-sided two-hole dry feeder, whereas the other provided one wet-dry feeder per pen. All pens provided water through gate-mounted water cups.

### Animals and Experimental Design

One thousand six hundred and eighty barrows and gilts (DNA 610E × DNA 241 F1, DNA Genetics, Columbus, NE) entered the barns after the nursery phase and were sorted into mixed-sex pens across two barns and fed a common diet for 49 days until the start of the experiment (day 0). Pigs were vaccinated against porcine circovirus type 2 and mycoplasma hyopneumoniae (Fostera Gold PCV MH, Zoetis, Parsippany, NJ) at the sow farm at the time of weaning. Upon the start of the experiment, pigs were assigned to a randomized complete block design by room within the barn and equalized by weight, with 12 pigs per pen within 20 replications. Pigs were housed at a commercial finishing research facility (Donald E. Orr Swine Farm, United Animal Health, Sheridan, IN) with four rooms with equal representation of each treatment in each room. Each room was blocked by starting body weight. Within each block, pens were randomly assigned to one of seven treatments (Table 1) in a nested arrangement with one control treatment that remained nutrient un-restricted for the duration of the study. In addition to the control treatment there were two restriction lengths: 3 (3 wk) or 6 wk (6 wk); and 3 lysine concentrations after restriction: feeding the same diet as control (100), a diet 10% greater than control (110), or a diet 20% higher than control (120) in terms of the standardized ileal digestible (SID) lysine to metabolizable energy ratio (Lys:ME) requirements for control pigs. The experiment consisted of three phases, phase 1: day 0-–21, phase 2: day 21–41, and phase 3: day 41 to market. The recovery period was phases 2 and 3 for 3 wk restricted pigs, and phase 3 for 6 wk restricted pigs.

**Table 1.** Experimental treatments and design by phase and corresponding days of the experiment

	Control	3 w k:100	3 wk:110	3 wk:120	6 wk:100	6 wk:110	6 wk:120
Phase 1 (0 to 21)	Control	Corn	Corn	Corn	Corn	Corn	Corn
Phase 2 (21 to 41)	Control	Control	Control + 10%	Control + 20%	Corn	Corn	Corn
Phase 3 (41 to Market)	Control	Control	Control + 10%	Control + 20%	Control	Control + 10%	Control + 20%

Control: corn/SBM finishing diets:100% Lys:ME.

(3 wk:100) 97% corn diet fed for 3 wk; then control: 100.

(3 wk:110) 97% corn diet fed for 3 wk; then control + 10% Lys:ME: 110.

(3wk:120) 97% corn diet fed for 3 wk; then control + 20% Lys:ME: 120.

(6 wk:100) 97% corn diet fed for 6 wk; then control: 100.

(6 wk:110) 97% corn diet fed for 6 wk; then control + 10% Lys:ME: 110.

(6 wk:120) 97% corn diet fed for 6 wk; then control + 20% Lys:ME: 120.

### Dietary Treatments

Pigs were fed a common diet (United Animal Health, Sheridan, IN) prior to the start of the experiment. At the start of the experiment, pigs assigned to a nutrient-restricted diet (Table 2) for either 3 or 6 weeks were provided a 97% corn diet which was a nutrient-restricted diet but met or exceeded vitamin and mineral requirements (NRC, 2012). This nutrient-restricted diet provided 0.48 vs. 2.26 SID Lys:ME for the nutrient adequate diet. The corn-holding diet only provided about 20% of the lysine requirements but was also restrictive in terms of other amino acids and had imbalanced amino acid ratios. After the respective restriction period, recovery diets consisted of either the same diet as control (100), a diet 10% greater than control (110), or a diet 20% greater than control (120) in terms of the SID Lys:ME.

**Table 2.** Diet information for experiment day 0 to market for pigs starting at 75.5 kg

Ingredient %	Phase 1 (days: 0 to 21)		Phase 2 (days: 21 to 41)				Phase 3 (days: 41-Market)		
	Control	Restricted	Control, 3 wk:100	3 wk:110	3 wk:120	Restricted	Control, 3 wk:100, 6 wk:100	3 wk:110, 6 wk:110	3 wk:120, 6 wk:120
Corn	77.25	97.45	81.10	78.2	74.98	97.78	81.76	78.65	76.09
SBM	17.68	–	14.07	17.02	20.32	–	13.45	16.64	19.23
Choice white grease	2.5	–	2.50	2.5	2.5	–	2.5	2.5	2.5
Limestone	0.81	0.86	0.75	0.74	0.74	0.79	0.75	0.75	0.74
Salt	0.62	0.63	0.62	0.62	0.62	0.63	0.62	0.62	0.62
Monocalcium phosphate, 21%	0.53	0.87	0.34	0.28	0.21	0.6	0.35	0.28	0.23
Vitamin and mineral premix^1^	0.2	0.2	0.20	0.2	0.2	0.2	0.2	0.2	0.2
L-Lysine	0.24	–	0.24	0.24	0.23	–	0.22	0.2	0.21
DL-Methionine	0.08	–	0.07	0.08	0.09	–	0.05	0.06	0.08
L-Threonine	0.09	–	0.10	0.11	0.11	–	0.09	0.09	0.1
Nat-P 2500 0.32# (0.125 aP)^2^	0.01	–	0.01	0.01	0.02	0.01	0.01	0.01	0.01
L-Tryptophan	–	–	0.01	0.01	–	–	0.01	–	–
Metabolizable energy, kcal/kg	3,272	3,137	3,280	3,282	3,285	3,148	3,278	3,278	3,282
Crude protein, %	13.77	6.6	12.37	13.55	14.85	6.62	12.08	13.34	14.39
Crude fat, %	5.37	3.13	5.4	5.38	5.36	3.14	5.41	5.39	5.37
Crude fiber, %	2.86	1.87	2.09	2.15	2.21	1.87	2.08	2.14	2.19
SID Lys: ME	2.26	0.48	2.01	2.21	2.41	0.48	1.91	2.1	2.29
SID Lys, %	0.74	0.15	0.66	0.73	0.79	0.15	0.63	0.69	0.75
SID SAA:Lys	0.58	1.38	0.58	0.58	0.58	1.38	0.58	0.58	0.58
SID Thr:Lys	0.65	1.12	0.68	0.68	0.68	1.12	0.68	0.68	0.68
SID Trp:Lys	0.18	0.29	0.19	0.19	0.19	0.29	0.19	0.19	0.19
SID Val:Lys	0.65	1.52	0.65	0.65	0.66	1.52	0.67	0.68	0.67
SID Ile:Lys	0.59	1.05	0.57	0.59	0.6	1.05	0.59	0.61	0.61
SID Leu:Lys	1.35	3.96	1.39	1.36	1.34	3.96	1.44	1.42	1.38
Available phosphorus, %	0.23	0.23	0.22	0.22	0.22	0.22	0.22	0.22	0.22
Added phytase, FTU/kg	202.2	57.8	311.2	338.3	368.7	200	305.8	335.3	359
Calcium, %	0.52	0.52	0.45	0.45	0.45	0.45	0.45	0.45	0.45
Total Ca:P	1.25	1.25	1.25	1.25	1.25	1.25	1.25	1.25	1.25
Sodium, %	0.25	0.25	0.25	0.25	0.25	0.25	0.25	0.25	0.25
Copper, mg/kg	16.35	14.2	15.83	16.2	16.61	14.13	15.76	16.15	16.48

^1^Vitamin and trace mineral; provided 4,008 IU vitamin A, 1,089 IU vitamin D, 27 IU vitamin E, 0.5 mg vitamin K, 34.6 mg niacin, 19 mg pantothenic acid, 4.4 mg riboflavin, 0.023 mg vitamin B12, 110 mg Zn (zinc sulfate), 98 mg Fe (iron sulfate), 40 mg Mn (manganese sulfate), 12 mg Cu (copper sulfate), and 0.3 mg Se (sodium selenite) per kilogram of diet.

^2^Released 0.078 and 0.156 aP, for 0.01% and 0.02% inclusions, respectively.

Diets were manufactured (Table 2) at the United Animal Health Donald E. Orr Swine Farm feed mill (Sheridan, IN). All diets were provided in mash form. Pigs had ad libitum access to water. Feed samples were collected from six different feeders weekly, for each dietary treatment in each phase. Feed samples were stored at −20 °C for subsequent analysis.

Feed samples were ground to 1 mm particle size using a Wiley Mill (Variable Speed Digital ED-5 Wiley Mill; Thomas Scientific, Swedesboro, NJ). Processed samples were homogenized and analyzed in duplicate for dry matter (DM; method 930.15 [AOAC, 2007]), acid-hydrolyzed ether extract (aEE; method 2003.06; [AOAC, 2007]), and ash (ASH; method 942.05; [AOAC, 2007]). Nitrogen and amino acid profile were determined (N; Agriculture Experiment Station Chemical Laboratories, Columbia, MO) and crude protein was calculated as N × 6.25. Gross energy was determined in duplicate using an isoperibolic bomb calorimeter (model 6200; Parr Instrument Co., Moline, IL). Benzoic acid (6,318 kcal GE/kg) was used as the standard for calibration and was determined to contain 6,317 ± 10 kcal GE/kg. Mycotoxin levels were tested at the United Animal Health research laboratory (Sheridan, IN; Romer AgraQuant Elisa) on the completed feed.

### Measurements

Pigs were tagged at birth with radio-frequency identification tags and the LeeO individual animal identification system (Prairie Systems, Spencer, IA) was used to record individual body weight (BW) at experiment days 0, 21, 41, and at market. Individual weights provide the ability to track variation between pens and BW was summed to calculate total pen weight and used to calculate average daily gain (ADG). Individual BW was also used to calculate pen standard deviation (SD) at the beginning and end of the experiment, thus the percent change is SD (change in SD, divided by the starting SD, multiplied by 100). Feed intake was monitored on a pen basis to calculate the average daily feed intake (ADFI) and gain-to-feed ratio (G:F). During the experiment, pigs showing signs of illness were treated in accordance with veterinary recommendations and farm procedures. Pigs not showing response to treatment within 48 h, were considered morbid and removed from the experiment. Morbidities and mortalities were weighed on the day they were removed from the experiment. Pig removal information was used during growth data analysis by adding the removal weight into the pen weight and the number of days the pig was on experiment for that phase added into total pig days.

To fully understand the compensation ability, all pigs in a pen were marketed (Tyson, Logansport, IN) on the same day when the pen averaged 131.5 kg ± 3.3. Pigs were tattooed with a unique number for pen identification once at the plant. All pigs were measured at the plant for carcass weight, loin depth, and back fat at the 10th rib. Lean percentage, yield percentage, carcass ADG, and carcass G:F were then calculated for each pen. The marketing strategy was to market each pen at a common weight (130 kg), however, some differences between treatments were observed due to the marketing schedule and weekend constraints. Therefore, days to market weight (130 kg) was calculated for each pen to understand marketing differences to a common weight.

A compensatory index value was used to evaluate the extent of catch-up growth of restricted treatments. Compensatory index value is calculated as the difference of weight between restricted treatments and control at the end of restriction (A) minus the difference of weight between restricted treatments and control treatments at the end of compensatory gain period (B), presented as a proportion over (A).

Compensatory index value, adapted from Wilson and Osbourn (1960):


$$Compensatory\;index\;\%=\frac{\left(A-B\right)}{A}\;x\;100$$

This equation is to represent the percentage of catch-up growth (Wilson and Osbourn, 1960), or the percentage of growth difference at the end of restriction from unrestricted pigs that was reclaimed by the end of the experiment. The weight difference for end of restriction (A) was calculated at days 21 and 41 for 3- and 6 wk restricted treatments, respectively. The end of the compensatory gain period was considered day 49, and market weights were back calculated using the ADG of each pen during the final period to establish their weight at day 49.

### Statistical Analysis

Growth performance data were analyzed as repeated measures using a generalized linear mixed model (Proc Glimmix, SAS 9.4, SAS Inst., Cary, NC). Pen was considered the experimental unit, with restriction length, lysine level nested within restriction level, phase, the interaction between phase and restriction, and the interaction between phase and lysine nested with restriction included as fixed effects. Block and room were included as random effects. The compound symmetry covariance structure was used for the repeated measures model according to the Bayesian information criterion. Overall (days 0-market) growth performance and carcass measurements were analyzed using mixed model methods (Proc mixed, SAS 9.4, SAS Inst., Cary, NC). Pen was the experimental unit, with restriction length and lysine level nested with restriction as fixed effects, and room and block as random effects. Market weight was used as a covariate for carcass data due to unintentional differences in market weight. When fixed effects were a significant source of variation (*P *< 0.05) least squares means were separated using pairwise *t*-tests (PDIFF option, SAS 9.4, SAS Inst., Cary, NC).

Testing of normality and homoscedasticity of the studentized residuals was done using the Univariate procedure. Unequal variance between phases was observed for repeated growth data, random residual by phase statement was used to correct for this variance. Statistical outliers were identified as data points occurring greater than 3 SDs of studentized residuals from the mean and were excluded from the analysis. Differences in total removals were insignificant and excluded from these data. Results were considered significant at *P* ≤ 0.05 and a trend at *P* > 0.05 and *P* ≤ 0.10.

## Results

### Analyzed Diets

Diet analysis (Table 3) indicated that the calculated SID lysine values from all phases and diets were slightly lower than the values measured and analyzed. Additionally, the analyzed fat content was consistently lower than the calculated fat content for all diets. The variation between the analyzed and calculated nutrients was steady overall diets and phases, which is expected. Mycotoxin analysis indicated that the complete diets were at safe level for finishing pig consumption.

**Table 3.** Analyzed nutritive contents for experiment day 0 to market (as-fed basis) for pigs starting at 75.5 kg

Nutrient	Phase 1 (days: 0 to 21)		Phase 2 (days: 21 to 41)				Phase 3 (days: 41 to Market)		
	Control	Restricted	Control, 3 wk:100	3 wk:110	3 wk:120	Restricted	Control, 3 wk:100, 6 wk:100	3 wk:110, 6 wk:110	3 wk:120, 6 wk:120
GE, kcal/kg	3,885	3,625	3,927	3,883	3,942	3,711	3,864	3,900	3,967
Fat, %	5.2	2.7	5.8	4.6	5.2	3.9	5.7	5.9	6.4
Dry matter, %	87.5	86.9	87.6	87.7	87.9	86.8	87.5	87.3	87.5
Ash, %	3.4	2.5	3.3	3.1	3.3	3.1	3.4	3.2	3.2
Crude protein, %^1^	13.2	6.5	11.2	13.1	13.4	7.7	12.2	13.5	14.1
Lysine, %	0.81	0.16	0.83	0.78	0.87	0.31	0.80	0.79	1.03
Methionine, %	0.24	0.11	0.21	0.22	0.24	0.15	0.22	0.24	0.31
Tryptophan, %	0.19	0.10	0.19	0.21	0.21	0.11	0.18	0.20	0.21
Threonine, %	0.51	0.22	0.50	0.54	0.54	0.28	0.51	0.52	0.64
Valine, %	0.63	0.30	0.59	0.61	0.66	0.36	0.57	0.60	0.77
Isoleucine, %	0.56	0.26	0.53	0.54	0.59	0.30	0.51	0.53	0.68
Leucine, %	1.21	0.80	1.16	1.16	1.26	0.80	1.11	1.17	1.38
Histidine, %	0.3 3	0.16	0.33	0.34	0.37	0.21	0.32	0.33	0.41
Phenylalanine, %	0.66	0.33	0.61	0.62	0.68	0.36	0.58	0.61	0.78
Aflatoxin, µg/kg	3.45	3.53	4.66	2.75	6.99	2.42	9.94	10.21	9.93
Vomitoxin, mg/kg	0.31	0.26	0.28	0.42	0.36	0.22	0.42	0.35	0.30
Fumonisin, mg/kg	0.17	0.76	0.18	0.38	0.00	0.12	0.00	0.01	0.05
Zearalenone, mg/kg	0.04	0.01	0.01	0.02	0.00	0.00	0.01	0.00	0.00

^1^Crude protein= %N × 6.25.

Diets with “3wk:” Three weeks on restricted diet.

Diets with “6wk:” Six weeks on restricted diet.

Diets with “:100”, fed 100% of Lys:ME of control pigs after restriction.

Diets with “:110”, fed 110% of Lys:ME of control pigs after restriction.

Diets with “:120”, fed 120% of Lys:ME of control pigs after restriction.

### Restriction Period

**Figure 1.**
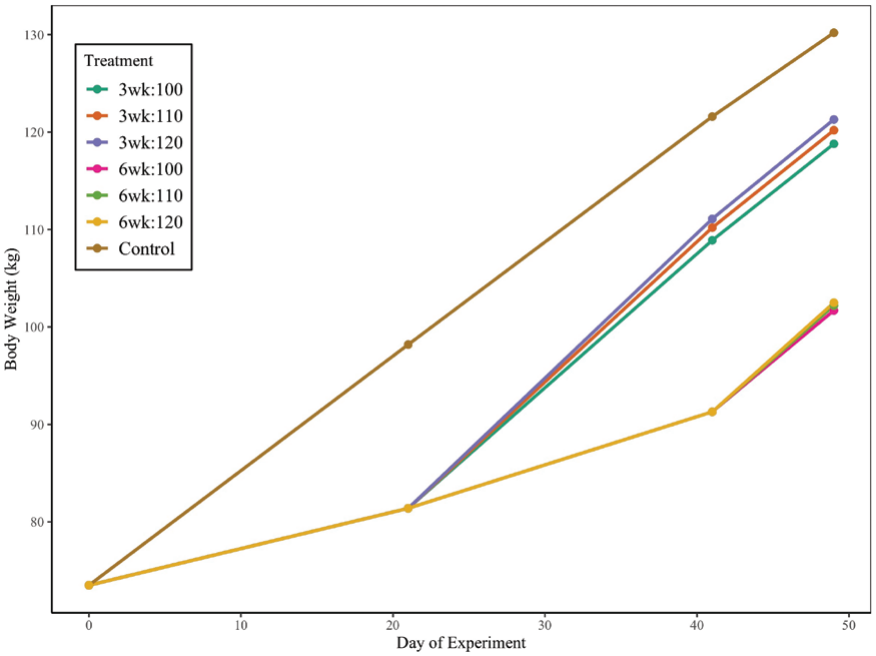
Body weight of pigs fed restricted diets for two different lengths, 3 or 6 weeks, and fed increasing Lysine:ME (100%, 110% or 120% Lys:ME of control) after restricted periods. Body weight is from day 0 to day 49 of experiment, when control pigs were market ready. Treatments 3 wk:100, 3 wk:110, and 3 wk:120 were removed from restriction on day 21. Treatments 6 wk:100, 6 wk:110, and 6 wk:120 were removed from restriction on day 41 of the experiment.

The body weight measurements can be visualized in Figure 1. Pigs started the experiment at an average BW of 73.5 ± 2.2 kg, the experiment lasted 62 ± 9 days, with an ending body weight of 131.5 ± 3.3 kg. From days 0–21 both the 3 and 6-wk restriction treatments were on the corn-holding diet and had decreased (*P *< 0.01; Table 4) BW, ADG, ADFI, and G:F. From days 21 to 41 the 3 wk restricted treatment was no longer restricted but the 6 wk treatment remained on the restricted diet and continued to have decreased (*P* < 0.01; Table 4) BW, ADG, ADFI, and G:F compared to control pigs.

**Table 4.** Growth performance with the main effects of lysine level after restriction and restriction length

Item	Lysine (rrestriction)							SEM	*P*-value	Restriction length			SEM	*P*-value	Day × lysine (restriction)
	Control	3 wk:100	3 wk:110	3 wk:120	6 wk:100	6 wk:110	6 wk:120			Control	3 wk	6 wk			*P*-value
**BW, kg**								0.99	0.83				0.99	<0.01	0.04
Day 0	73.5	73.5	73.5	73.5	73.5	73.5	73.5			73.5	73.5	73.5			
Day 21^1^	98.2^a^	81.4^b^	81.4^b^	81.4^b^	81.4^b^	81.4^b^	81.4^b^			98.2^x^	81.4^y^	81.4^y^			
Day 41^2^	121.6^a^	108.9^c^	110.2^b^	111.1^b^	91.3^d^	91.3^d^	91.3^d^			121.6^x^	110.1^y^	91.3^z^			
Market^3^	132.2^ab^	131.1^abc^	130.1^bc^	129.8^c^	133.0^a^	132.4^ab^	131.7^abc^			132.2^x^	130.3^y^	132.4^x^			
**ADG, kg**								0.03	<0.01				0.03	<0.01	0.08
Days 0-21^1^	1.2^a^	0.4^b^	0.4^b^	0.4^b^	0.4^b^	0.4^b^	0.4^b^			1.2^x^	0.4^y^	0.4^y^			
Days 21-41^2^	1.2^d^	1.4^c^	1.4^b^	1.5^a^	0.5^e^	0.5^e^	0.5^e^			1.2^y^	1.4^x^	0.5^z^			
Days 41-market	1.1^e^	1.2^d^	1.3^d^	1.3^cd^	1.3^c^	1.4^b^	1.4^a^			1.1^z^	1.3^y^	1.4^x^			
**ADFI, kg**								0.05	0.70				0.05	<0.01	0.99
Days 0-21^1^	2.9^a^	2.5^b^	2.5^b^	2.5^b^	2.5^b^	2.5^b^	2.5^b^			2.9^x^	2.5^y^	2.5^y^			
Days 21-41^2^	3.4^a^	3.4^a^	3.3^a^	3.4^a^	2.8^b^	2.8^b^	2.8^b^			3.4^x^	3.4^x^	2.8^y^			
Days 41-market	3.3^b^	3.5^a^	3.4^ab^	3.4^a^	3.4^ab^	3.4^ab^	3.4^a^			3.3^y^	3.4^x^	3.4^x^			
**G:F**								0.01	<0.01				0.01	<0.01	<0.01
Days 0-21^1^	0.41^a^	0.15^b^	0.15^b^	0.15^b^	0.15^b^	0.15^b^	0.15^b^			0.4^x^	0.1^y^	0.1^y^			
Days 21-41^2^	0.34^d^	0.41^c^	0.43^b^	0.44^a^	0.17^e^	0.17^e^	0.17^e^			0.3^y^	0.4^x^	0.2^z^			
Days 41-market	0.32^e^	0.36^d^	0.36^d^	0.37^d^	0.38^c^	0.40^b^	0.42^a^			0.3^z^	0.4^y^	0.4^x^			

Day and day × restriction significant (*P* < 001).

^1^3 wk restriction period.

^2^6 wk restriction period.

^3^Marketing occurred when pen met common weight.

Within a row data without common superscripts differ, abcd denotes differences for the main effect of lysine (restriction) vs. xyzdenotes differences for the effect of restriction length.

### Compensatory Period

As the 3 wk restricted treatment was returned to nutrient-adequate diets they showed increased (*P* < 0.01) ADG and G:F, with similar (*P* > 0.1) ADFI compared to control pigs, indicating compensatory growth. Additionally, within the 3 wk restricted treatment, there was an effect (*P* < 0.05; Table 4) of the lysine level in the recovery diet (100, 110, and 120) for BW, ADG, and G:F for the first 3 weeks of recovery. For days 41 to market 6wk restricted pigs were removed from restriction and fed their respective recovery diet. Results indicated that 3- and 6 wk restricted treatments showed increased (*P* < 0.01) ADG and G:F over control pigs during the recovery period. A response in ADG and G:F due to the increased lysine concentration in recovery was observed for 6 wk restricted treatments for this final period but not for 3 wk restricted treatments. These indicate that the length of the restriction has an impact either way, but the increased lysine concentration seems to only be beneficial within the first 3 wk of recovery. For the overall experiment (Table 5) restricted pigs still had lower ADG, ADFI, and G:F compared to unrestricted pigs (*P* < 0.01). However, pigs on 120% Lys:ME recovery diets performed better than 100% Lys:ME recovery diets within their respective restriction treatment.

**Table 5.** Cumulative experiment parameters with the main effects of lysine level after restriction and restriction length

Parameter	Lysine (restriction)							SEM	*P*-value	Restriction length				
	Control	3 wk:100	3 wk:110	3 wk:120	6 wk:100	6 wk:110	6 wk:120			Control	3 wk	6 wk	SEM	*P*-value
ADG, kg	1.17^a^	0.98^c^	0.99^cb^	1.01^b^	0.81^e^	0.83^de^	0.84^d^	0.014	0.03	1.17^x^	0.99^y^	0.82^z^	0.014	<0.01
ADFI, kg	3.17^a^	3.08^b^	3.07^b^	3.05^bc^	3.00^cd^	2.96^d^	2.94^d^	0.036	0.30	3.17^x^	3.07^y^	2.97^z^	0.036	<0.01
G:F, kg	0.37^a^	0.32^c^	0.32^c^	0.33^b^	0.27^f^	0.28^e^	0.29^d^	0.002	<0.01	0.37^x^	0.32^y^	0.28^z^	0.002	<0.01
Days to 130 kg ^2^	49^e^	58^c^	56^cd^	55^d^	70^a^	69^ab^	68^b^	1.1	<0.01	49^x^	57^y^	69^z^	1.1	<0.01
Day 49, BW, kg^1^^,^^2^	130.2^a^	118.8^c^	120.2^bc^	121.3^b^	101.7^d^	102.2^d^	102.5^d^	1.07	<0.01	130.2^x^	120.1^y^	102.1^z^	1.07	<0.01
Beginning SD	15.7	17.2	16.7	16.9	18	17.1	16.6	0.88	0.77	15.7	16.9	17.3	0.88	0.28
Ending SD	22.3^b^	30.4^a^	25.2^b^	26.1^b^	32.2^a^	31.6^a^	30.4^a^	1.69	0.08	22.3^z^	27.2^y^	31.4^x^	1.69	<0.01
Change in SD, %^3^	42.6^b^	76.9^a^	52.6^b^	55.3^b^	80.2^a^	88.4^a^	79.6^a^	6.19	0.01	42.6^z^	61.6^y^	82.7^x^	6.04	<0.01
Compensatory Index, %^4^	–	33.7^c^	41.7^b^	48.4^a^	5.8^c^	7.4^c^	8.3^c^	3.76	<0.01	–	41.3^x^	7.1^y^	3.35	<0.01

^1^Day 49 was the first day of marketing for the control treatment.

^2^ADG for each pen in the marketing period was used to calculate days to 130 kg.

^3^Calculated as: ((change in SD)/(starting SD) × 100).

^4^Calculated as the ratio of the difference between weight at the end of restriction period (day 21 for 3 wk restricted pigs, day 41 for 6 wk restricted pigs) and compensatory growth period (when the control pigs met market weight: day 49).

SD = standard deviation.

Within a row data without common superscripts differ, abcd denotes differences for the main effect of lysine (restriction) vs. xyz denotes differences for the effect of restriction length.

### Marketing

The first group of control pigs reached the target market weight by day 49 of the experiment. Due to the method of marketing at a common weight instead of on a common day, pigs had a similar market weight. The longer the pigs were restricted, the more time it took for them to reach 130 kg (*P* < 0.01; Table 5). Days to market differed (*P* < 0.01) within each restriction treatment based on the lysine level in recovery, with a decrease in days to market as the lysine level in the recovery diet increased. Through back calculations to day 49 using the ADG of each treatment, it was evident that the restricted pigs were unable to achieve full compensation. Moreover, the pigs subjected to longer periods of restriction had the lowest body weight.

### Compensation

A compensatory index value was used to determine how well each treatment was able to catch up to the control treatment. Results indicate that 3 wk restricted pigs were able to compensate better than 6 wk restricted pigs, additionally, the 3:120 treatment was able to compensate better (*P* < 0.01; Table 5) than any other treatment. It is worth noting that the percentage change in standard deviation (Table 5) within a pen was impacted by both restriction (*P* < 0.01) and the lysine level (*P *= 0.01) fed after restriction.

### Carcass Measurements

Market weight was statistically different across treatments, due to marketing schedule constraints; therefore, it was used as a covariate for carcass parameters. Regardless of holding duration or lysine level in recovery, when compared to control pigs, pigs on the corn holding diets had decreased (*P* < 0.01; Table 6) carcass weight, loin depth, lean percentage, yield percentage, carcass ADG, carcass G:F, and increased back fat. Increasing the lysine level during the recovery period within the 6 wk restriction treatment increased (*P* < 0.01; Table 6) loin depth and G:F, and had a positive impact on lean percentage for both the 3 wk and 6 wk restricted treatments.

**Table 6.** Carcass characteristics with the main effects of lysine level after restriction and restriction length

Parameter	Control	Lysine (restriction)						SEM	*P-value*	Restriction			SEM	*P-value*
		3 wk:100	3 wk:110	3 wk:120	6 wk:100	6 wk:110	6 wk:120			0	3 wk	6 wk		
Shipped weight, kg	132.2^ab^	131.0^bc^	130.1^c^	129.7^c^	133.3^a^	132.6^ab^	132.7^ab^	0.78	0.66	132.2^x^	130.27^y^	132.9^x^	0.78	<0.01
Carcass weight, kg^1^	98.8^a^	97.7^bc^	98.1^ab^	97.8^bc^	97.1^c^	97.2^bc^	97.4^bc^	0.38	0.82	98.8^x^	97.9^y^	97.2^z^	0.38	<0.01
Backfat, cm^1^	1.5^c^	1.6^b^	1.5^c^	1.5^c^	1.7^a^	1.6^ab^	1.7^a^	0.03	0.07	1.5^z^	1.6^y^	1.7^x^	0.03	<0.01
Loin depth, cm^1^	7.3^a^	6.9^b^	7.1^b^	7.1^b^	6.5^d^	6.6^c^	6.7^c^	0.05	0.01	7.3^x^	7.0^y^	6.6^z^	0.05	<0.01
Lean, %^1^	56.9^a^	56.1^c^	56.3^bc^	56.3^b^	55.1^e^	55.4^d^	55.4^d^	0.13	<0.01	56.9^x^	56.2^y^	55.3^z^	0.13	<0.01
Yield, %^1^,^3^	75.1^a^	74.3^bc^	74.6^ab^	74.4^bc^	73.8^c^	73.9^bc^	74.1^bc^	0.29	0.84	75.12^x^	74.4^y^	73.9^z^	0.29	<0.01
Carcass ADG, kg^1^^,^^2^	0.8^a^	0.7^b^	0.7^b^	0.7^b^	0.6^c^	0.6^c^	0.6^c^	0.01	0.24	0.8^x^	0.7^y^	0.6^z^	0.01	<0.01
Carcass G:F ^1^^,^^2^	0.3^a^	0.2^b^	0.2^b^	0.2^b^	0.2^d^	0.2^c^	0.2^c^	0.003	<0.01	0.3^x^	0.2^y^	0.2^z^	0.003	<0.01

^1^Shipped weight included in model as covariate.

^2^Calculated from experiment D0-market on a carcass basis.

^3^Yield calculated using live weight measured at the barn and the hot carcass weight from the plant (Tyson Logansport, IN) within a row data without common superscripts differ, abcd denotes differences for the main effect of lysine (restriction) vs. xyz denotes differences for the effect of restriction length.

## Discussion

In this study, the corn-holding diet reduced growth at a comparable rate to Gabler et al. (2020) with a similar starting body weight and same method of nutrient restriction. In this study, the growth rate slowed by 58% and 67% for 6 and 3 wk restriction periods, respectively, compared to unrestricted pigs. In combination with reduced growth, pigs fed corn holding (restricted diets) experienced decreased feed intake, resulting in poor feed efficiency.

Several studies did not indicate reductions in feed intake during similar high corn restriction methods (Gabler et al., 2020; Helm et al., 2021; Rao et al., 2021). However, with the large statistical power in the present study, it was found that pigs on a corn-holding diet reduced their feed intake. The reduction in feed intake using such diets may be due to the imbalance in amino acids (NRC, 2012). The restricted diets in the current study had high ratios of SAA (methionine + cystine), threonine, tryptophan, isoleucine, and leucine to lysine compared to the ideal protein concept (NRC, 2012). Studies have shown manipulation of amino acid ratios such as increasing methionine to lysine (Edmonds and Smith, 2021), can effectively reduce feed intake, such as seen in the current experiment. Other amino acid imbalances such as high tryptophan (Edmonds and Baker, 1987) and leucine (Gatnau et. al., 1995) compared to lysine can also reduce eating behavior and growth. These imbalances may have been a reason feed intake decreased in such feeding scenarios.

After the respective nutrient restriction period, pigs were fed a nutrient-unrestricted diet or a diet above lysine requirements until they weighed 130 kg, for a recovery period. During this period, restricted pigs had increased ADG and G:F compared to control pigs. Pigs restricted for 3-wk grew 16.7% faster than the control pigs, and pigs allowed the highest Lys:ME diet grew 10% faster than the pigs allowed the same diet as control. Similarly, it was shown that 6 wk restricted pigs had a 27.3% higher ADG than control with the highest Lys:ME diet also performing 10% better. This experiment was unable to evaluate how long the increased ADG in the compensatory gain period would last because of marketing, but Skiba et al., (2001) reported the increase in growth performance to last 4–6 wk after the change in diet for smaller pigs.

The increase in ADG in the recovery period was not accompanied by an increase in feed intake over control pigs, as appetite in this period is controlled by the body fa t and leptin stores deposited in the restriction period (O’Connell et al., 2006; Reynolds and O’Doherty, 2006; Martínez-Ramírez et al., 2009). Thus, compensatory gain for pigs on previous nutrient restriction, specifically lysine restriction, is due to feed efficiency, as agreed by Menagat et al. (2020). This is initiated by improved nitrogen utilization in the recovery period (Fabian et al., 2004; Reynolds and O’Doherty, 2006; Ishida et al., 2011; Ishida et al., 2012). A consistent improvement in lysine utilization was observed after periods of restriction over unrestricted pigs (Whang et al., 2003; Fabian et al., 2004; O’Connell et al., 2006; Ishida et al., 2012; Cloutier et al., 2016). Therefore, Whang et al., (2003) suggested lysine requirements may be higher in a recovery period, which was evaluated in the present study.

An increase in lysine concentration in recovery diets over recommendations for control pigs, increased the ADG and G:F during the compensatory growth period in nutrient-restricted pigs and accentuates compensatory gain. The 120% treatment was able to compensate faster and reach market weight 2 to 3 d earlier than those fed the same as control after restriction. During a recovery period, nitrogen utilization as a percentage of intake and protein deposition increases (O’Connell et al., 2006), and coinciding with periods of increased protein deposition, lysine requirement also increases (Williams et al., 1997), aligning with the notion that lysine requirements may be higher during a period of compensatory growth (Whang et al., 2003). Nevertheless, the increased performance from increased supplementation of lysine in the recovery period does not last the whole time. The 120% treatment only over-performs the 100% treatment for the first 3-wk of recovery, as shown by the 3 wk restricted treatments. Lysine requirements in recovery may be higher early in recovery and decrease to a normal level later in the period, suggesting that a phased approach may be needed. While the increase in lysine concentration did not increase feed intake, it did increase feed costs which must be considered when deciding w hat recovery diet to feed after nutrient restriction.

A similar phased approach of decreasing lysine as the pig grows is common in pigs fed adequate diets the entire growing period. Notably, the pigs fed the restrictive 97% corn diet were lower in body weight than the age-equivalent pigs in the control treatment. Understanding that amino acid requirements for lower BW pigs are higher than those of greater body weight, it is not surprising that pigs after a holding period require a higher amino acid concentration than the age-equivalent control treatment simply because of the body weight differences.

Allowing pigs to reach market weight (within a pen) allowed for the true market weight carcass characteristics to be evaluated after a period of restriction and recovery. This also makes it difficult to compare to other experiments that are marketed on a common day regardless of the live weight of the pig. In this experiment, carcass quality was influenced by both the restriction length and the lysine concentration after restriction. A similar increase in backfat, thus, decrease in loin depth, due to corn-holding diets was observed by Rao et al., (2021). Several reports show an increase in backfat from the corn-only diets or low crude protein diets without the allowance of a recovery period, in support of the hypothesis that the increased backfat was created during the restriction period (Kerr et al., 1995; Ruusunen et al., 2007; Morazán et al., 2015; Helm et al., 2021). The establishment of a thicker backfat is mostly due to energy repartitioning to lipid deposition, rather than lean tissue, in the absence of adequate amino acids (de Greef et al., 1992; Kerr et al., 1995; Kamalakar et al., 2009; Martínez-Ramírez et al., 2009; Suárez-Belloch et al., 2015), and because of this, pigs under previous lysine restriction engage in compensatory efforts during the recovery phase to replenish body protein stores. This supports the idea by Skiba (2005) that growth after the removal of a lysine restriction is mostly protein deposition, whereas removal of intake restriction results in more lipid deposition and internal organ weight. Unfortunately, meat quality was unable to be tested in this experiment, however, pigs on a low dietary protein diet have previously been shown to have increased intramuscular fat (Cisneros et al., 1996a,b) and lighter color (Kerr et al., 1995; Ruusunen et Al. 2007). Ultimately, we do not know if this is similar in pigs allowed a recovery period.

The compensatory index (CI) value was used to evaluate how well each treatment caught up to the control treatment after recovery. A CI value of 100 means that restricted pigs were able to recover 100% of the weight difference during a recovery period, which is very rarely observed (Hornick et al., 2000). As reported, no restricted treatment was able to fully compensate by the time that the control pigs were ready for market. However, pigs restricted for 3 weeks were able to compensate over 40% of the weight difference by the time unrestricted pigs went to market, conversely, the 6wk restricted pigs were only able to recover about 7% of the weight difference. This is largely due to the control pigs going to market only 1-wk after the 6 wk restricted treatment started their recovery diets and using the overall calculated ADG for their recovery period to back-calculate the weight at day 49. This does not account for a possible early spike in ADG within the first week of recovery. It would have been more beneficial to record the body weight of these pigs at the same time control pigs were going to market. The 3 wk:120 treatment was able to compensate about 15% more than those in the 3 wk:100 treatments, further supporting that nutrient requirements may be higher in recovery after a period of restriction.

The ability to individually weigh each pig allowed the tracking of variation within a pen, measured by standard deviation. Pigs fed holding diets have higher variation, indicating that individual pigs may respond differently to a lysine restrictive and subsequent recovery period.

The 97% corn diet was successful at slowing the growth of pigs starting at an average body weight of 73.5 kg, with time to market increasing as the time on the restrictive diet increases. A shorter period of restriction, or increased lysine concentration in recovery, increased the level of compensatory gain. When formulating diets for a recovery period, for the purpose of capturing the most compensatory gain, the lysine level should be based on the body weight at the end of restriction and potentially increased beyond that level. In terms of carcass measurements, the carcass merit of pigs placed on the 97% corn diet did not fully return to the level observed in unrestricted pigs. However, an improvement was observed when the lysine of the recovery diet was increased.
